# Treatment patterns and adherence to lipid-lowering therapy, LDL-C control, clinical outcomes, and healthcare resource utilization in treated patients with hypercholesterolemia at high cardiovascular risk in Israel: a retrospective database study

**DOI:** 10.3389/fcvm.2026.1672698

**Published:** 2026-04-30

**Authors:** Matan Azulay, Naama Yekutiel, Moran Elboim, Moshe Hoshen, Sivan Gazit, Oren Shavit

**Affiliations:** 1 Novartis Israel Ltd., Tel Aviv, Israel; 2 Maccabi Research and Innovation Center, Maccabi Healthcare Services, Tel Aviv, Israel

**Keywords:** cardiovascular disease outcomes, healthcare resource utilization, hypercholesterolemia, lipid-lowering therapy, low-density lipoprotein cholesterol, treatment adherence

## Abstract

**Aims:**

In this retrospective study, we analyzed data from patients with hypercholesterolemia to describe treatment patterns, adherence, low-density lipoprotein cholesterol (LDL-C) control, cardiovascular outcomes, and healthcare resource utilization among treated patients in a real-world setting in Israel.

**Methods:**

Deidentified data from the medical records of patients with atherosclerotic cardiovascular disease (ASCVD), ASCVD-risk equivalent (ASCVD-RE), and familial hypercholesterolemia (FH) treated with lipid-lowering therapy (LLT) between 2013 and 2019 were analyzed. LDL-C values were based on routine laboratory reporting in Maccabi Healthcare Services and were calculated using the Friedewald equation during the study period. Controlled LDL-C was defined as LDL-C < 70 mg/dL for ASCVD patients and <100 mg/dL for ASCVD-RE and FH patients. Adherence to LLTs was assessed using the proportion of days covered (PDC), and associations with cardiovascular outcomes and healthcare resource utilization were evaluated.

**Results:**

A total of 15,258 patients with hypercholesterolemia (FH [7.0%], ASCVD [33.3%], and ASCVD-RE [59.7%]) were identified. Most of them (>90%) received statin monotherapy, while 2.9% received a combination of a statin and other LLTs. High adherence to LLTs (PDC ≥ 80%) was achieved by 54.1% of ASCVD patients, followed by 35.3% of ASCVD-RE patients and 17.7% of FH patients. Combination therapy with high-intensity statins (HIS) plus ezetimibe was associated with lower first postinitiation LDL-C levels in the ASCVD and ASCVD-RE groups, with 47.6% and 52.0% of patients, respectively, achieving LDL-C < 55 mg/dL; Among FH patients, 30.8% achieved LDL-C levels of 70–100 mg/dL with HIS monotherapy. A PDC ≥ 80% was significantly associated with a longer time to percutaneous coronary intervention and CABG procedures and a longer time to death in both the ASCVD and ASCVD-RE groups. During the study period, LDL-C targets were achieved by 61.1% of ASCVD patients. LDL-C < 100 mg/dL was reached by 79.9% of ASCVD-RE patients and 33.5% of FH patients.

**Conclusions:**

In this large, treated real-world cohort, substantial gaps in LDL-C control and treatment adherence were observed across high-risk populations. Higher adherence to lipid-lowering therapy was associated with better LDL-C control and longer time to selected clinical outcomes; however, these findings should be interpreted as associations rather than evidence of causality, given the observational study design.

**Translational perspective:**

In this real-world cohort, higher adherence to lipid-lowering therapy (PDC ≥ 80%) was associated with better LDL-C control and more favorable cardiovascular outcome patterns. These findings highlight the potential importance of sustained treatment adherence as a key determinant of lipid control in routine clinical practice and suggest that strategies supporting long-term adherence may contribute to improved cardiovascular risk management in high-risk populations.

## 1 Introduction

Hypercholesterolemia is a major modifiable contributor to atherosclerotic cardiovascular disease (ASCVD), and elevated levels of cholesterol-rich apolipoprotein B (ApoB)-containing lipoproteins play a central causal role in atherogenesis (1, 2). low-density lipoprotein (LDL) is the principal driver of the initiation and progression of atherosclerotic plaques, and high LDL-cholesterol (LDL-C) levels are associated with increased mortality from ischemic heart disease and stroke, which together account for 3.6 million deaths globally every year (3).

The relationship between LDL-C and ASCVD is well established across epidemiologic, experimental, genetic, and randomized clinical trials (1). Consequently, lowering LDL-C is a central strategy in cardiovascular prevention and may halt the progression of atherosclerotic plaques while reducing the incidence of ASCVD (4, 5).

The efficacy of statins in lowering cholesterol levels is well established. They provide an effective and well-tolerated method for the prevention of cardiovascular (CV) events in adults with hyperlipidemia (6) and thus represent one of the most investigated drug classes in terms of treatment adherence (7). However, studies have shown poor adherence and relatively low long-term persistence with statins, with approximately half of patients discontinuing therapy within several months (8, 9). A retrospective study assessed persistence with statin therapy using the proportion of days covered (PDC) and its correlation with LDL-C levels, suggesting that PDC is associated with LDL-C reduction (10). For individuals requiring further LDL-C lowering according to the level of risk, a combination of statin and non-statin lipid-lowering therapy (LLT), such as ezetimibe and/or PCSK9 inhibitors, is recommended (9).

Current European guidelines recommend intensive LDL-C lowering in high-risk populations, with treatment targets of <70 mg/dL for high-risk patients and <55 mg/dL for very high-risk patients (11). Nevertheless, achieving these targets in routine clinical practice remains challenging. This challenge is particularly relevant in high-risk populations, including patients with established ASCVD, individuals with ASCVD-risk equivalent conditions, and patients with familial hypercholesterolemia (FH) (12), a genetic disorder characterized by markedly elevated LDL-C levels and a substantially increased lifetime risk of cardiovascular disease (6, 13).

In Israel, real-world data describing lipid-lowering treatment patterns and LDL-C goal attainment in specific high-risk groups have begun to emerge; however, these studies have generally been limited to particular clinical settings and relatively short follow-up periods. Comprehensive, integrated analyses that concurrently examine treatment patterns, long-term adherence, LDL-C control, cardiovascular outcomes, and healthcare resource utilization within the same large real-world high-risk population remain scarce (14). Improved understanding of these factors may help identify gaps in care and inform strategies to optimize cardiovascular risk management and resource utilization within healthcare systems.

The aim of this study was to describe treatment patterns, adherence to lipid-lowering therapy, LDL-C goal attainment, cardiovascular outcomes, and healthcare resource utilization among patients with ASCVD, ASCVD-risk equivalent conditions, and familial hypercholesterolemia using a large real-world healthcare database from Israel. We further examined whether greater adherence to lipid-lowering therapy was associated with improved LDL-C control and more favorable outcome patterns.

## 2 Methods

### 2.1 Study design

This was a retrospective, non-interventional cohort study designed to assess treatment patterns, clinical burden, and healthcare resource utilization associated with hypercholesterolemia in Israel among patients with established ASCVD (ASCVD), ASCVD-risk equivalent (ASCVD-RE), and FH patients with probable and confirmed diagnosis. Patients with a medical record of ASCVD and hypercholesterolemia were classified as ASCVD patients. The study was based on the following objectives: (1) to estimate the prevalence of ASCVD, ASCVD-RE, and FH among patients treated with LLTs; (2) to describe their baseline demographics and clinical characteristics; (3) to evaluate their adherence to LLT over time; (4) to evaluate the association between treatment adherence and controlled LDL-C levels, as well as the association between treatment adherence and CV events of interest [i.e., myocardial infarction (MI), stroke, revascularization, and all-cause death] over time; and (5) to assess HCRU.

The study was conducted in partnership with Maccabi Healthcare Services (MHS), a nationwide healthcare payer-provider organization in Israel, and in accordance with the International Council for Harmonisation (ICH) Good Clinical Practice (GCP) guidelines and the Declaration of Helsinki: Ethical Principles for Medical Research Involving Human Subjects. The study was approved by the MHS Institutional Review Committee Board (IRB number 0098-21-MHS). Due to its retrospective design and the deidentified nature of the data, the requirement for informed consent was waived by the IRB. All analyses were conducted using anonymized data extracted from the MHS electronic database. As safety reporting was not planned per study protocol, data on adverse events were not routinely collected.

### 2.2 Data source and access

Deidentified patient data, including demographics, laboratory results, pharmacy prescriptions, and medication purchase records, were accessed, collected, and extracted by MHS researchers from the MHS electronic database, which contains longitudinal clinical and administrative data from a population of >2.7 million members. The data underlying this article cannot be shared publicly due to their confidential and proprietary nature.

MHS comprises several automatically generated registries, including a CVD registry (15). The database applies the International Classification of Diseases, Ninth Revision, Clinical Modification (ICD-9-CM) coding system. LLT medications were coded according to the Israeli coding system, with translations to ATC coding system where available. Procedures were coded using the current procedural terminology (CPT) code (16) (see Supplementary Appendix A).

### 2.3 Study population

Patients treated with LLT between 1 January 2013 and 31 December 2019 were included in the study if they met all of the following criteria:

1. Patients had a first purchase of a statin or another LLT within the abovementioned 7-year period. The date of the first lipid-lowering medication purchase was defined as the index date. Follow-up time was calculated from the index date until the end of follow-up, death, or treatment discontinuation, whichever occurred first.
2. Continuous enrollment in MHS for 1 year prior to the index date.
3. Age ≥18 years at the index date.

For the analyses of treatment adherence, only patients with at least 1 year of follow-up were included. For outcome analyses, only patients with at least 5 years of continuous enrollment and follow-up (or death) were included. Patients with cancer (included in the existing MHS cancer registry and the Israeli National Cancer Registry) were excluded from the study (17).

#### 2.3.1 Patient groups

The study population was divided into three patient groups as follows:

1. FH group—patients with a diagnosis of FH or those who met the make early diagnosis to prevent early deaths (MED-PED) criteria: (18)
  (a) Aged <20 years and LDL-C ≥ 270 mg/dL.
  (b) Aged 20–29 years and LDL-C ≥ 290 mg/dL.
  (c) Aged 30–39 years and LDL-C ≥ 340 mg/dL.
  (d) Aged ≥40 years and LDL-C ≥ 360 mg/dL.

Patients without an FH diagnosis who met the MED-PED criteria for the first time at ≥65 years or those diagnosed with autoimmune, liver, thyroid diseases, and chronic kidney disease (CKD) (eGFR <60) were not included in the FH group (18).

2. ASCVD group—patients from the remaining population (non-FH) who had experienced ASCVD events, including MI, cerebrovascular accident (CVA), transient ischemic attack (TIA), peripheral vascular disease, or had undergone percutaneous coronary intervention (PCI)/coronary artery bypass graft (CABG) procedures before the index date. ICD-9 code/CPT codes were used to define ASCVD events (Table 1).
3. ASCVD-RE group—patients from the remaining population (neither FH nor ASCVD) who were defined as at risk by a SCORE chart value of ≥5% (19) or who had DM (>10 years) or CKD (stage B, C, or D) prior to LLT initiation.

Patients receiving statin therapy who did not meet the criteria for any of the three groups were excluded from the study.

**Table 1:** ASCVD definition.

Acute coronary syndrome		
Myocardial infarction (MI)	ICD-9 codes	MI registry 410
Unstable angina (UA)	ICD-9 codes	UA registry 411.1
Stable angina (SA)	ICD-9 codes	SA registry 413.9
Revascularization^a^		
Percutaneous coronary intervention (PCI)	CPT code	PCI registries 92980, 92982,92984
Coronary artery bypass graft (CABG)	CPT code	CABG registries 33510
Others		
Stroke (cerebrovascular accident/event)	ICD-9 codes	CVA registries 430, 431, 432.x, 433.x, 434.x, 435.x, 436
Transient ischemic attack (TIA)	ICD-9 codes	TIA registry 435.9
Peripheral artery disease (PAD)	ICD-9 codes	PAD registry 443.9
Additional morbidity		
Diabetes	ICD-9 codes	Diabetes registry
Hypertension	ICD-9 codes	Hypertension registry
Chronic kidney disease (CKD)	ICD-9 codes	CKD registry
Congest heart failure (CHF)	ICD-9 codes	CHF registry
Atrial fibrillation (AF)	ICD-9 codes	AF registry
Obstructive sleep apnea (OSA)	ICD-9/MSH codes: 780.51, 780.53, 780.57, corresponding internal Maccabi codes	
Major depressive disorder (MDD)	ICD-9/MSH codes: 296.2, 296.3, 296.30, corresponding internal Maccabi codes	
Left ventricular hypertrophy (LVH)	ICD-9/MSH codes: 429.3, corresponding internal Maccabi codes	
Nonalcoholic fatty liver (NAFL)	ICD-9/MSH codes: 571.8, corresponding internal Maccabi codes	

a To define recent ASCVD, ICD-9, and CPT codes were used for all conditions to avoid losing patients, as the registry captured only the first event, which may be older than 12 months.

### 2.4 Study variables and measurements

#### 2.4.1 Baseline characteristics

Baseline characteristics used to describe the patient groups included demographic and lifestyle factors such as sex, age, socioeconomic status (SES, defined based on the individual's residential address), smoking status (ever/never), BMI (20), chronic comorbidities, and concomitant medications. Neighborhoods were stratified into 10 distinct SES levels by the Israeli Central Bureau of Statistics, initially analyzed as a continuous variable, and then categorized into three levels (low 1–4, medium 5–7, and high 8–10). Baseline LDL-C levels were calculated as the mean of all measurements obtained during the 12 months prior to the index date. All baseline characteristics were categorized using standard cut-points.

#### 2.4.2 Chronic comorbidities and concomitant medications

Chronic comorbidities, including DM, hypertension, CKD, heart failure, atrial fibrillation (AF), major depressive disorder (MDD), obstructive sleep apnea (OSA), left ventricular hypertrophy (LVH), and non-alcoholic fatty liver disease (NAFLD), were identified throughout each patient's medical history up to the index date and were defined using validated MHS registries where available. The following clinical conditions were defined using relevant ICD-9 or MHS codes: MDD, OSA, LVH, and NAFLD. Clinical and laboratory results were collected for systolic blood pressure (SBP), triglycerides, lipoprotein(a), and total cholesterol. PCI and CABG procedures were defined using CPT codes (see Supplementary Appendix A).

Medications dispensed within the 12 months prior to the index date were recorded and analyzed per patient group. A full list of concomitant medications, categorized by ATC codes, is provided in Supplementary Appendix A. Statin therapies were classified as high-, moderate-, or low-intensity based on their dose and degree of LDL reduction (21). LLT medications were grouped as follows: (1) high-intensity statins (HIS), including both high- and moderate-intensity doses of statins; (2) non-HIS, including low-intensity doses of statins; (3) ezetimibe; (4) HIS+ezetimibe; (5) non-HIS+ezetimibe; (6) PCSK9 inhibitors (monotherapy or in combination with statins or ezetimibe); and (7) fibrates (monotherapy or in combination with statins or ezetimibe).

### 2.5 LDL-C measurements

LDL-C concentrations were obtained from lipid profile measurements recorded in the MHS database. During the study period, LDL-C values were calculated using the Friedewald equation as part of routine laboratory reporting in Maccabi Healthcare Services. All lipid measurements were performed as part of standard clinical care.

### 2.6 LDL-C treatment goals

During the study, LDL-C goals reflected the European guideline recommendations in effect during the study period (2013–2019) (22). Accordingly, controlled LDL-C levels were defined as < 70 mg/dL for ASCVD patients and <100 mg/dL for ASCVD-RE and FH patients.

### 2.7 Treatment adherence

Patient adherence to LLT medications was assessed using the PDC), defined as the proportion of days during which each patient had access to the medication. PDC was calculated for each LLT until the end of follow-up, or death, or treatment switching, whichever occurred first. Patients were considered adherent to the LLT medications if PDC ≥ 80%. The first LDL-C measurement after LLT initiation for patients with PDC ≥ 80%, both first and mean LDL-C levels during each LLT medication course for patients with PDC ≥ 80% and PDC < 80%, and LDL-C levels across the five main LLT medications (HIS, non-HIS, ezetimibe, HIS+ezetimibe, and non-HIS+ezetimibe) were monitored throughout the study to assess patient adherence to and persistence with each LLT and to evaluate the possibility of achieving controlled LDL-C levels within 3–6 months of initiating a new LLT. This calculation was repeated for each patient and for each treatment course. Because adherence was assessed for each LLT course, individual patients could contribute more than one treatment episode during the study period. Each treatment course was therefore analyzed as a separate observation.

### 2.8 Study outcomes

CVD diagnoses during the study period, including MI, stroke, TIA, and peripheral artery disease (PAD), were obtained from hospital discharge records. Events of interest, including MI, unstable/stable angina, and revascularization (PCI and CABG), were defined using the respective ASCVD ICD-9 codes (see Supplementary Appendix A). Mortality was assessed using all-cause mortality data from the National Insurance Institute.

The association between PDC ≥ 80% and study outcomes was assessed among patients treated with LLT medications by each treatment group (each patient could be included in multiple LLT groups) for at least 6 months, as follows:

1. Time to first MI, CVA, and TIA events after LLT initiation in the ASCVD-RE group. In the FH group, only a small number of CV events were recorded; therefore, since only primary events were captured in the database, this analysis was not applicable to both FH and ASCVD groups.
2. Time to PCI or CABG procedure and time to death after LLT initiation for both ASCVD and ASCVD-RE groups.

### 2.9 Healthcare resource utilization

The association between HCRU and PDC was assessed across all patients to characterize disease-related healthcare utilization based on the following parameters evaluated per patient-year (PY) and/or per patient: (1) any hospitalization and the number of hospitalizations; (2) duration of hospitalization, measured as by the number of hospitalization days; (3) number of emergency room (ER) visits; (4) number of visits to a primary care physician (PCP); and (5) any visit to a cardiologist.

### 2.10 Statistical analyses

All analyses were conducted using IBM SPSS version 29 (IBM SPSS, Armonk, NY, USA). Descriptive statistics were presented as numbers (percentages), means ± SDs, or medians, as appropriate. Comparisons of proportions or means across groups were performed using the chi-square test for categorical variables and the *t*-test for continuous variables, or the Kruskal–Wallis test, as appropriate. Cox proportional hazards analysis was used to determine risk factors for mortality for the time interval from the procedure until the end of follow-up. The number of hospitalizations, ER visits, and PCP visits were presented per PY of follow-up.

Multiple logistic regression models were used to describe the association between PDC ≥ 80% and controlled LDL-C separately for each study group, adjusting for predefined confounders including age, sex, socioeconomic status, baseline lipid levels, chronic comorbidities, and concomitant cardiovascular medications. Associations were expressed as odds ratios (ORs) and corresponding 95% confidence intervals (CIs). Multivariable Cox proportional hazards regression, adjusted for relevant confounders, was used to estimate the association between PDC ≥ 80% and study outcomes (first MI/CVA, PCI/CABG, death, and their composite) after at least 6 months of LLT medication use. Associations were expressed as hazard ratios (HRs) with corresponding 95% CIs. A two-tailed *p*-value of ≤0.05 was considered statistically significant. Analyses were performed using available data from the MHS database.

## 3 Results

### 3.1 Study population

A total of 118,449 MHS patients who purchased LLT between 2013 and 2019 were identified and screened for inclusion in the study. Of these, 91.2% received only statins as their first LLT during the study period. Overall, 74,987 patients met all inclusion criteria and comprised the eligible study population (see Supplementary Tables S1a,b).

The final study population, defined as patients with hypercholesterolemia who had LDL-C measurements in the year prior to the index date, comprised 15,258 patients. Of these, 1,062 (7.0%) patients were allocated to the FH group, 5,083 (33.3%) to the ASCVD group, and 9,113 (59.7%) to the ASCVD-RE group (see Supplementary Table S2).

### 3.2 Baseline characteristics

The FH group comprised 56.7% men. Most patients (51.9%) had medium SES, and 26.7% had a BMI ≥ 30 kg/m2. The most common comorbidities (recorded in >10% of the patients) were DM and hypertension. CKD was recorded in 5.6% of patients. All concomitant medications in this patient group were recorded by fewer than 10% of patients, with the highest proportions (6.8%) receiving angiotensin-converting enzyme (ACE) inhibitors and aspirin (6.4%). The mean (SD) LDL-C and total cholesterol levels were 208.3 (50.1) and 302.9 (59.3) mg/dL, respectively. The mean (SD) triglyceride level was 230.8 (264.6) mg/dL (Table 2).

**Table 2:** Baseline characteristics of the study population by patient groups.

Patient characteristics	FH *n* = 1,062	ASCVD *n* = 5,083	ASCVD-RE *n* = 9,113
Age (years), mean (SD)	37.1 (11.1)	60.1 (11.7)	65.4 (11.6)
Male, *n* (%)	460 (56.7)	3,728 (73.3)	4,513 (49.5)
SES^c^, *n* (%)			
Low (1–4)	233 (22.0)	1,044 (20.6)	1,847 (20.3)
Medium (5–7)	550 (51.9)	2,654 (52.3)	4,859 (53.4)
High (8–10)	277 (26.1)	1,378 (27.1)	2,386 (26.2)
BMI^d^ (kg/m^2^), *n* (%)			
<25	404 (39.4)	1,287 (25.9)	2,286 (25.6)
25–29.99	348 (33.9)	2,181 (43.9)	3,633 (40.8)
≥30	274 (26.7)	1,505 (30.3)	2,995 (33.6)
Smoking ever, *n* (%)	243 (22.9)	1,137 (22.4)	918 (10.1)
Comorbidities, *n* (%)			
Diabetes mellitus	111 (10.5)	717 (14.1)	2,891 (31.7)
Hypertension	132 (12.4)	2,410 (47.4)	4,854 (53.3)
CKD	60 (5.6)	1,023 (20.1)	5,655 (62.1)
CHF	1 (0.1)	140 (2.8)	106 (1.2)
AF	1 (0.1)	383 (7.5)	354 (3.9)
MDD	15 (1.4)	111 (2.2)	205 (2.2)
OSA	49 (4.6)	513 (10.1)	688 (7.5)
LVH	14 (1.3)	140 (2.8)	218 (2.4)
NAFL	1 (0.1)	49 (1.0)	44 (0.5)
Medication within 12 months before the index date, *n* (%)			
Beta blockers	41 (3.9)	2,608 (51.3)	2,145 (23.5)
ACE inhibitors	72 (6.8)	2,159 (42.5)	2,370 (26.0)
ARBs	46 (4.3)	917 (18.0)	1,999 (21.9)
P2Y12 inhibitors	6 (0.6)	2,434 (47.9)	366 (4.0)
Aldosterone agonists	2 (0.2)	286 (5.6)	187 (2.1)
Aspirin	68 (6.4)	2,730 (53.7)	2,261 (24.8)
SGLT-2 inhibitors	4 (0.4)	28 (0.6)	101 (1.1)
Laboratory tests within 12 months before the index date, mean (SD)			
Systolic BP	121.1 (15.1)	131.3 (18.6)	133.7 (17.8)
LDL^a^ (mg/dL)	*n* = 889 208.3 (50.1)	*n* = 4,889 126.3 (31.0)	*n* = 8,742 144.9 (34.1)
Triglyceride^b^ (mg/dL)	230.8 (264.6)	143.6 (74.7)	143.8 (75.5)
Total cholesterol (mg/dL)	302.9 (59.3)	201.0 (38.0)	225.6 (40.2)

Between-group differences analyzed using the chi-square test, Student’s *t*-test, and/or the Kruskal–Wallis test, as appropriate, and were statistically significant for all baseline parameters [*p* < 0.001 (*p* = 0.020 for NAFL)], except for SES (*p* = 0.488) and MDD (*p* = 0.207).

ACE inhibitors, angiotensin-converting enzyme inhibitors; AF, atrial fibrillation; ARBs, angiotensin II receptor blockers; ASCVD, patients with atherosclerotic cardiovascular disease; ASCVD-RE, ASCVD-risk equivalent patients; BMI, body mass index; BP, blood pressure; FH, familial hypercholesterolemia; LDL, low-density lipoprotein; LVH, left ventricular hypertrophy; MDD, major depressive disorder; NAFL, non-alcoholic fatty liver; OSA, obstructive sleep apnea; P2Y12 inhibitors, purinergic receptor P2Y12 protein (G-protein-coupled) inhibitors; SES, socioeconomic status; SGLT-2 inhibitors, sodium–glucose cotransporter-2 inhibitors.

a Only patients for whom LDL could be measured.

b Not significant between ASCVD and ASCVD-RE patient groups.

c Patients with missing data (two patients in the FH group, seven patients in the ASCVD group, and 21 patients in the ASCVD-RE group).

d Patients with missing data (36 patients in the FH group, 109 patients in the ASCVD group, and 199 patients in the ASCVD-RE group).

The ASCVD group comprised 73.3% men. Most patients (52.3%) had medium SES, and 30.3% had BMI ≥ 30 kg/m^2^. The most common comorbidities (recorded in >10% of the patients) were DM, hypertension, CKD, and OSA. The most commonly used concomitant medications (recorded in >10% of the patients) were beta blockers, ACE inhibitors, angiotensin II receptor blockers (ARBs), purinergic receptor P2Y12 (G-protein-coupled) inhibitors, and aspirin. The mean (SD) LDL-C and total cholesterol levels were 126.3 (31.0) and 201.0 (38.0) mg/dL, respectively. The mean (SD) triglyceride level was 143.6 (74.7) mg/dL (Table 2).

The ASCVD-RE group comprised 49.5% men. Most patients (53.4%) had medium SES, and 33.6% had BMI ≥ 30 kg/m^2^. The most common comorbidities (recorded in >10% of the patients) were DM, hypertension, and CKD. The most commonly used concomitant medications (recorded in >10% of the patients) were beta blockers, ACE inhibitors, ARBs, and aspirin. The mean (SD) LDL-C and total cholesterol levels were 144.9 (34.1) mg/dL and 225.6 (40.2) mg/dL, respectively. The mean (SD) triglyceride level was 143.8 (75.5) mg/dL (Table 2).

### 3.3 Adherence to treatment

#### 3.3.1 Adherence to lipid-lowering therapies

##### 3.3.1.1 Adherence to PCSK9 inhibitor and PCSK9 inhibitor combination therapies

Only small numbers of patients from the three study groups received PCSK9 inhibitor monotherapy (eight, 56, and 22 patients in the FH, ASCVD, and ASCVD-RE groups, respectively), most of whom achieved PDC ≥ 80% (75.0%, 67.9%, and 59.1%, respectively). Similarly, only a few patients were treated with PSCK9i in combination with ezetimibe (two patients each in the FH and ASCVD-RE groups, and three patients in the ASCVD group) or with PSCK9i in combination with statins (three patients in the FH group and 13 patients in the ASCVD group); all of these patients had PDC ≥ 80%. Due to the small number of patients in each group receiving fibrates or fibrate–statin combination therapy, a relatively large proportion of patients on these LLTs had PDC ≥ 80% across all three patient groups, with the highest number observed in the ASCVD group [23 (69.7%) patients]. Data on patient adherence to all LLT medication types used during the study are provided in Supplementary Table S3.

Due to the small number of patients receiving PCSK9 inhibitors as monotherapy or in combination with statins or ezetimibe, as well as those receiving fibrates alone or in combination with statins, the available data were insufficient; therefore, these LLTs were not included in the analyses of LDL-C levels or study outcomes.

##### 3.3.1.2 Adherence to the five main LLT medications

**Figure 1:**
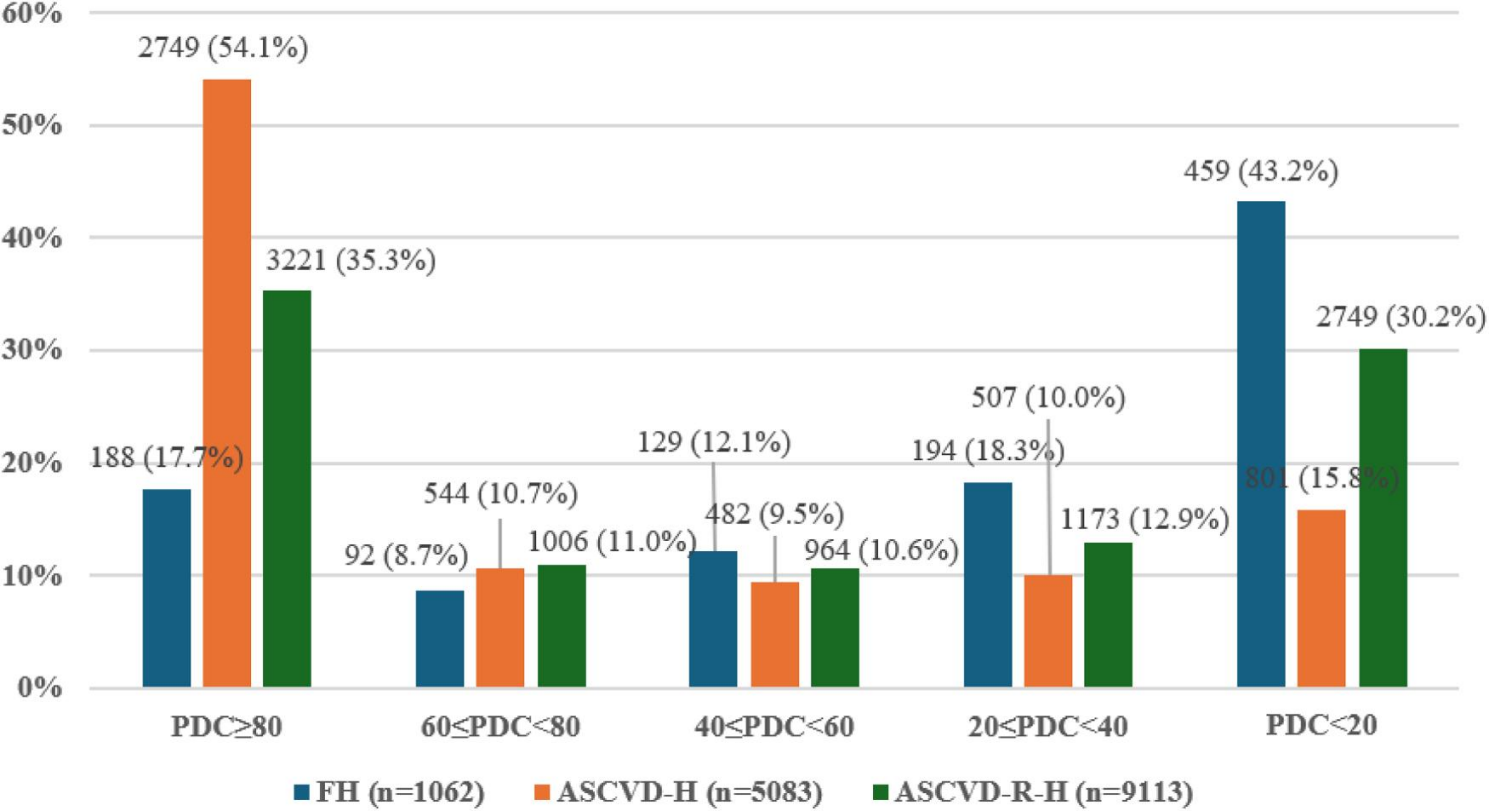
Adherence to the five main LLT medications by patient group and PDC. ASCVD, patients with atherosclerotic cardiovascular disease; ASCVD-RE, ASCVD-risk equivalent patients; FH, familial hypercholesterolemia; HIS, high-intensity statin; LLT, lipid-lowering therapy; PDC, proportion of days covered. Between-group differences analyzed using the Kruskal–Wallis test were statistically significant for PDC ≥ 80% and PDC < 80% (*p* < 0.001).

High adherence to the five main LLT medications (PDC ≥ 80%) was achieved by 2,749 (54.1%) patients in the ASCVD group, followed by 3,221 (35.3%) patients in the ASCVD-RE group and 188 (17.7%) patients in the FH group. The lowest adherence (PDC < 20%) was observed in the FH group (43.2% of patients). Adherence levels of 60% ≤ PDC < 80% were similar in the ASCVD and ASCVD-RE groups (reached by 544 [10.7%] and 1,006 [11.0%] patients, respectively) and higher than in the FH group [reached by 92 (8.7%) patients] (Figure 1).

#### 3.3.2 First LDL-C levels after initiation of lipid-lowering therapy

Among the 1,062 FH patients, PDC ≥ 80% was achieved by 89 (8.4%) patients on HIS monotherapy, 142 (17.4%) on non-HIS monotherapy, 9 (52.9%) on ezetimibe monotherapy, 43 (53.8%) on HIS+ezetimibe, and 35 (52.2%) on non-HIS+ezetimibe therapy. The mean first LDL-C levels were >100 mg/dL with all LLTs, except for the HIS+ezetimibe combination, under which FH patients reached the first mean (SD) LDL-C of 96.5 (40.3) mg/dL. The highest proportion of patients (31.6%) achieved an LDL-C reduction in the range of 70–100 mg/dL with non-HIS + ezetimibe. LDL-C < 55 mg/dL was recorded for 9.6% of patients on HIS monotherapy, 2.1% on non-HIS monotherapy, and 11.8% on HIS + ezetimibe therapy.

Of the 5,083 patients in the ASCVD group, PDC ≥ 80% was achieved by 2,082 (40.9%) patients on HIS monotherapy, 1,261 (43.7%) on non-HIS monotherapy, 62 (50.8%) on ezetimibe monotherapy, 387 (80.6%) on HIS+ezetimibe, and 257 (66.1%) on non-HIS+ezetimibe. The mean (SD) first LDL-C levels were <100 mg/dL, with the lowest value [59.6 (23.7) mg/dL] observed with HIS+ezetimibe. The highest proportion of patients (36.0%) receiving non-HIS monotherapy had first LDL-C levels in the range of 70–100 mg/dL, while 38.9% of patients on ezetimibe monotherapy had first LDL-C ≥ 100 mg/dL. The highest proportions of patients on HIS monotherapy, HIS+ezetimibe, and non-HIS+ ezetimibe (41.7%, 47.6%, and 43.0%, respectively) had first LDL-C < 55 mg/dL. This level was reached by 21.9% of patients on non-HIS monotherapy and 8.3% of those on ezetimibe monotherapy.

Of the 9,113 patients in the ASCVD-RE group, PDC ≥ 80% was achieved by 958 (10.5%) patients on HIS monotherapy, 2,735 (33.9%) on non-HIS monotherapy, 94 (45.9%) on ezetimibe monotherapy, 176 (73.9%) on HIS+ezetimibe, and 255 (60.6%) on non-HIS+ezetimibe. The mean (SD) first LDL-C levels were <100 mg/dL across all LLTs, except for ezetimibe monotherapy, where a mild reduction was observed [103.6 (33.1) mg/dL], likely due to the low number of patients receiving this treatment. The highest proportions of patients on HIS monotherapy (31.7%), non-HIS monotherapy (42.9%), and non-HIS+ezetimibe (29.4%) had first LDL-C values in the range of 70–100 mg/dL, while 50% of the patients on ezetimibe monotherapy had first LDL-C ≥ 100 mg/dL. LDL-C levels <55 mg/dL were achieved by most patients (52.0%) treated with HIS+ezetimibe, as well as by 28.6% of those on HIS monotherapy, 10.6% on non-HIS monotherapy, 5.8% on ezetimibe monotherapy, and 28.7% on non-HIS+ezetimibe therapy.

All data on the first LDL-C measurements after LLT initiation for patients with PDC ≥ 80%, stratified by the LDL-C range, are presented in Supplementary Table S4.

#### 3.3.3 LDL-C levels during each lipid-lowering therapy

Each of the five main LLTs showed a sustainable effect on LDL-C levels among patients with PDC ≥ 80%. Mean LDL-C values measured during each LLT course across the three patient groups were highly comparable with those measured after LLT initiation. In addition, the highest proportions of patients in each study group achieved the same LDL-C ranges with the respective LLT medications. Exceptions were observed in the FH patients, 44.0% of whom achieved LDL-C levels in the range of 70–100 mg/dL when treated with HIS+ezetimibe, and in ASCVD-RE patients, who achieved LDL-C levels in the range of 55–70 mg/dL when treated with non-HIS + ezetimibe.

Data on LDL-C levels during each LLT for patients with PDC ≥ 80% and PDC < 80%, stratified by LDL-C range and treatment group, are presented in Supplementary Tables S5, S6.

#### 3.3.4 LDL-C levels during the study by PDC

##### 3.3.4.1 LDL-C levels for patients with PDC ≥ 80%

Overall, 188 (17.7%) patients with FH, 2,749 (54.1%) patients with ASCVD, and 3,221 (35.3%) patients with ASCVD-RE achieved PDC ≥ 80% during the study.

In the FH group, the mean (SD) LDL-C level was 114.3 (34.4) mg/dL, with 66.5% of patients having LDL-C ≥ 100 mg/dL, indicating that these patients failed to reach the treatment goal. In contrast, ASCVD-RE patients had a mean (SD) LDL-C level of 81.8 (23.8) mg/dL, with 79.9% achieving LDL-C <100 mg/dL. These patients had a higher proportion of achieving treatment goals. In the ASCVD group, the mean (SD) LDL-C level was 67.5 (19.6) mg/dL, with 61.2% of patients achieving LDL-C < 70 mg/dL (Table 3).

**Table 3:** LDL-C values during the study for the five main lipid-lowering therapies by patient group and proportion of days covered.

LLT	Patients with LDL-C measurements (mg/dL), *n* (%)	FH *n* = 1,062	ASCVD *n* = 5,083	ASCVD-RE *n* = 9,113
HIS, non-HIS, ezetimibe, HIS + ezetimibe, and non-HIS + ezetimibe	PDC ≥ 80	188 (17.7)	2,749 (54.1)	3,221 (35.3)
	LDL-C, mean (SD)	*n* = 167 114.3 (34.4)	*n* = 2,694 67.5 (19.6)	*n* = 3,122 81.8 (23.8)
	LDL-C < 55	6 (3.6)	720 (26.7)	347 (11.1)
	LDL-C 55–70	7 (4.2)	927 (34.4)	678 (21.7)
	LDL-C 70–100	43 (25.7)	876 (32.5)	1,471 (47.1)
	LDL-C ≥ 100	111 (66.5)	171 (6.3)	626 (20.1)
	PDC < 80	874	2,334	5,892
	LDL, mean (SD)	*n* = 781 156.5 (48.0)	*n* = 2,248 100.0 (31.1)	*n* = 5,582 117.0 (31.9)
	LDL-C < 55	15 (1.9)	128 (5.7)	112 (2.0)
	LDL-C 55–70	13 (1.7)	267 (11.9)	231 (4.1)
	LDL-C 70–100	50 (6.4)	777 (34.6)	1,408 (25.2)
	LDL-C ≥ 100	703 (90.0)	1,076 (47.9)	3,831 (68.6)

Within-group differences for PDC ≥ 80% vs. PDC < 80% per LLT (analyzed using Student’s *t*-test) were statistically significant (*p* < 0.001) for all groups, except for the FH patients who received ezetimibe monotherapy and non-HIS+ezetimibe combination therapy (see Supplementary Table S6e).

Between-group comparisons are performed using the chi-square test, and the difference between mean LDL-C levels of the PDC ≥ 80% group and the PDC < 80% group was statistically significant (*p* < 0.001).

ASCVD, patients with atherosclerotic cardiovascular disease; ASCVD-RE, ASCVD-risk equivalent patients; FH, familial hypercholesterolemia; HIS, high-intensity statin; LDL-C, low-density lipoprotein-cholesterol; LLT, lipid-lowering therapy; PDC, proportion of days covered.

##### 3.3.4.2 LDL-C levels for patients with PDC <80%

Overall, 874 (82.3%) patients with FH, 2,334 (45.9%) patients with ASCVD, and 5,892 (64.6%) patients with ASCVD-RE had PDC <80% during the study.

The mean (SD) LDL-C levels were ≥100 mg/dL across all groups [156.5 (48.0) mg/dL] in the FH group, 100.0 (31.1) mg/dL in the ASCVD group, and 117.0 (31.9) mg/dL in the ASCVD-RE group]. The highest proportion of patients in each study group (90.0% in the FH group, 47.9% in the ASCVD group, and 68.6% in the ASCVD-RE group) had LDL-C ≥ 100 mg/dL. During the study, LDL-C < 55 mg/dL was achieved by 1.9% of FH patients, 5.7% of ASCVD patients, and 2.0% of ASCVD-RE patients (Table 3).

#### 3.3.5 Association between treatment adherence and controlled LDL-C

In the FH group, none of the analyzed confounders were significantly associated with controlled LDL-C, except for female sex. However, multivariable logistic regression indication associations with age, total cholesterol levels, hypertension, DM, the use of beta blockers and P2Y12 inhibitors, and comorbid OSA (Table 4).

**Table 4:** Factors associated with controlled LDL-C levels.

Patient characteristics	FH^b^ *n* = 1,062		ASCVD *n* = 5,083		ASCVD-RE *n* = 9,113	
	OR (95% CI)	*p*-Value	OR (95% CI)	*p*-Value	OR (95% CI)	*p*-Value
PDC ≥ 80%	3.37 (0.83, 13.72)	0.090	8.57 (7.34, 10.02)	<0.001	10.1 (8.58, 11.88)	<0.001
Age at the index date	1.03 (0.96, 1.10)	0.464	1.0 (1, 1.01)	0.609	1.01 (1.0, 1.02)	0.002
Sex (female = 1)	0.90 (0.13, 0.63)	0.015	0.57 (0.48, 0.68)	<0.001	0.66 (0.56, 0.78)	<0.001
Socioeconomic status						
Low	1 (ref)		1 (ref)		1 (ref)	
Medium	0.58 (0.11, 3.08)	0.520	0.98 (0.81, 1.18)	0.810	1.09 (0.90, 1.32)	0.378
High	0.46 (0.06, 3.65)	0.464	1.41 (1.15, 1.73)	0.001	1.21 (0.97, 1.50)	0.089
BMI > 30 kg/m^2^^a^	0.64 (0.11, 3.60)	0.613	1.11 (0.95, 1.30)	0.194	1.21 (1.03, 1.42)	0.017
Diabetes mellitus^a^	2.23 (0.29, 17.14)	0.441	1.52 (1.23, 1.87)	<0.001	1.74 (1.48, 2.05)	<0.001
Hypertension^a^	2.09 (0.27, 16.31)	0.483	0.97 (0.83, 1.13)	0.704	1.08 (0.91, 1.28)	0.375
Systolic BP^a^	0.97 (0.91, 1.03)	0.287	1.0 (1.0, 1.01)	0.583	1.0 (0.99, 1.01)	0.168
LDL-C^a^	0.97 (0.93, 1.02)	0.305	0.99 (0.99, 0.99)	<0.001	0.98 (0.97, 0.99)	<0.001
Total cholesterol	1.01 (0.97, 1.05)	0.648	0.99 (0.98, 0.99)	<0.001	0.99 (0.98, 0.99)	<0.001
Aspirin	0.26 (0.01, 10.95)	0.482	0.96 (0.83, 1.10)	0.529	1.20 (1.02, 1.40)	0.027
Beta blockers^a^	2.52 (0.03, 207.4)	0.682	1.54 (1.34, 1.78)	<0.001	1.29 (1.09, 1.52)	0.003
P2Y12 inhibitors^a^	6.93 (0.89, 54.1)	0.065	1.65 (1.43, 1.91)	<0.001	1.51 (1.12, 2.03)	0.006
OSA	1.22 (0.10, 14.37)	0.874	0.80 (0.63, 1.01)	0.059	1.32 (1.03, 1.68)	0.027
SGLT-2 inhibitors^a^	–	–	1.40 (0.53, 3.70)	0.498	0.84 (0.48, 1.47)	0.542
MDD^a^	–	–	1.14 (0.70, 1.85)	0.595	1.03 (0.62, 1.72)	0.915
NAFL^a^	–	–	1.30 (0.86, 1.96)	0.217	1.23 (0.82, 1.84)	0.329
LVH^a^	–	–	0.93 (0.46, 1.85)	0.828	1.50 (0.65, 3.48)	0.342

Data were analyzed using a multiple logistic regression model with the low SES set as a reference and female = 1.

SGLT-2, depression, NAFL, and LVH were not included in the analysis of the FH group.

ASCVD, patients with atherosclerotic cardiovascular disease; ASCVD-RE, ASCVD-risk equivalent patients; BMI, body mass index; BP, blood pressure; FH, familial hypercholesterolemia; LDL-C, low-density lipoprotein cholesterol; LVH, left ventricular hypertrophy; MDD, major depressive disorder; NAFL, non-alcoholic fatty liver; OR, odds ratio; OSA, obstructive sleep apnea; P2Y12 inhibitors, purinergic receptor P2Y12 protein (G-protein-coupled) inhibitors; PDC, proportion of days covered; SES, socioeconomic status; SGLT-2 inhibitors, sodium–glucose cotransporter-2 inhibitors.

a Patient characteristic prior to the index date.

b Only a few FH patients had LDL-C < 100 mg/dL.

In the ASCVD and ASCVD-RE groups, PDC ≥ 80% was significantly associated with controlled LDL-C levels. In the ASCVD group, baseline LDL-C, female sex, higher SES, total cholesterol values, DM, and the use of beta blockers and P2Y12 inhibitors were also significantly associated with controlled LDL-C levels. The independence of covariates was tested by analyzing the correlation matrix and variance inflation factor (VIF). Correlations were <0.35 in all cases and VIF values were <2, thus confirming the inclusion of all variables in the models. Although not statistically significant, positive correlations were observed with age, medium SES, BMI > 30 kg/m^2^, systolic BP, use of aspirin and SGLT-2 inhibitors, and comorbidities including hypertension, OSA, MDD, non-alcoholic fatty liver (NAFL), and LVH. In the ASCVD-RE group, additional factors significantly associated with controlled LDL-C levels included age, female sex, BMI > 30 kg/m^2^, total cholesterol, and the use of aspirin, beta blockers, and P2Y12 inhibitors. Among the analyzed comorbidities, OSA and DM showed significant correlations. Although not statistically significant, associations with controlled LDL-C in this group were also observed for SES, systolic BP, use of SGLT-2 inhibitors, and comorbidities including hypertension, MDD, NAFL, and LVH (Table 4).

### 3.4 Study outcomes

Multivariable regression analysis, adjusted for relative confounders (age, sex, and chronic comorbidities), was used to estimate the association between PDC ≥ 80% and study outcomes, including first MI, CVA, TIA, PCI, and CABG procedures, and death, after at least 6 months of LLT therapy.

#### 3.4.1 Myocardial infarction, cardiovascular accident, and transient ischemic attack

In the ASCVD-RE group, age, SES, systolic BP, and the use of P2Y12 inhibitors were significantly associated with the occurrence of MI, CVA, and TIA. In addition, BMI > 30 kg/m^2^, hypertension, smoking, LDL-C levels, use of aspirin, and comorbidities such as OSA, depression, and NAFL were found to correlate with these cardiovascular outcomes, although not significantly. Data analysis did not indicate association between PDC ≥ 80% and time to first MI, CVA, and TIA in this study group (Table 5).

**Table 5:** Factors associated with time to first MI, CVA, and TIA event after LLT initiation for the ASCVD-RE group.

Patient characteristics	ASCVD-RE *n* = 9,113	
	HR (95% CI)	*p*-Value
PDC ≥ 80%	0.87 (0.53, 1.43)	0.576
Age at the index date	1.04 (1.01, 1.06)	0.004
Sex (female = 1)	0.84 (0.51, 1.39)	0.507
Socioeconomic status		
Low	1 (ref)	
Medium	2.30 (1.03, 5.16)	0.043
High	3.32 (1.43, 7.72)	0.005
BMI > 30^a^	1.16 (0.70, 1.93)	0.555
Diabetes mellitus^a^	0.93 (0.54, 1.60)	0.794
Hypertension^a^	1.09 (0.63, 1.87)	0.759
Ever smoking	1.79 (0.87, 3.67)	0.114
Systolic BP^a^	1.02 (1.0, 1.03)	0.025
LDL-C^a^	1.0 (0.99, 1.01)	0.634
Total cholesterol	0.99 (0.98, 1.01)	0.284
Aspirin	1.27 (0.78, 2.08)	0.337
Beta blockers^a^	0.86 (0.49, 1.49)	0.585
P2Y12 inhibitors^a^	2.94 (1.42, 6.07)	0.004
OSA	1.07 (0.45, 2.52)	0.879
MDD^a^	1.97 (0.61, 6.32)	0.257
NAFL^a^	1.19 (0.29, 4.90)	0.811

Only a few events were recorded in the FH group; therefore, this group was not included in the analysis. The data were analyzed only for primary events, so the analysis was not conducted for the ASCVD group.

Data were analyzed using a multivariable Cox regression model with the low SES set as a reference and female = 1.

ASCVD-RE, ASCVD-risk equivalent patients; BMI, body mass index; BP, blood pressure; FH, familial hypercholesterolemia; LDL-C, low-density lipoprotein cholesterol; MDD, major depressive disorder; NAFL, non-alcoholic fatty liver; OR, odds ratio; OSA, obstructive sleep apnea; P2Y12 inhibitors, purinergic receptor P2Y12 protein (G-protein-coupled) inhibitors; PDC, proportion of days covered; SES, socioeconomic status.

a Patient characteristic before the index date.

#### 3.4.2 Percutaneous coronary intervention and coronary artery bypass graft procedures

PDC ≥80% was significantly associated with time to PCI and CABG procedures in both the ASCVD and ASCVD-RE groups.

In the ASCVD group, confounders significantly associated with time to PCI and CABG included female sex, DM, and the use of beta blockers and P2Y12 inhibitors. Additional patient characteristics associated with these procedures, although not significantly, included higher SES, BMI > 30 kg/m^2^, hypertension, smoking, systolic BP, and total cholesterol. In the ASCVD-RE group, age, female sex, systolic BP, comorbid DM, and the use of beta blockers and P2Y12 inhibitors were significantly associated with time to PCI and CABG. In addition, higher SES, LDL-C, total cholesterol levels, and MDD were also associated with time to PCI/CABG, although not significantly.

Factors associated with time to PCI and CABG procedures after LLT initiation in the ASCVD and ASCVD-RE groups are presented in Supplementary Table S7.

#### 3.4.3 Death

PDC ≥80% was significantly associated with a longer time to death in both the ASCVD and ASCVD-RE groups. Patient characteristics significantly associated with time to death for ASCVD patients were age, higher SES, smoking, use of beta blockers, and comorbidities such as diabetes, hypertension, and LVH.

Additional confounders, although not significantly associated, included BMI > 30 kg/m^2^, SBP, total cholesterol levels, use of P2Y12 inhibitors, and diagnosis of MDD and NAFL.

For the ASCVD-RE patients, age, female sex, SES, smoking, use of beta blockers and P2Y12 inhibitors, and comorbidities such as DM, hypertension, and MDD were significantly associated with time to death.

Systolic BP, total cholesterol levels, OSA, and NAFL diseases were not significantly associated with the time to a fatal event (Table 6).

**Table 6:** Factors associated with time to death after LLT initiation for the ASCVD and ASCVD-RE groups.

Patient characteristics	ASCVD *n* = 5,083		ASCVD-RE *n* = 9,113	
	HR (95% CI)	*p*-Value	HR (95% CI)	*p*-Value
PDC ≥ 80%	0.67 (0.51, 0.89)	0.006	0.63 (0.50, 0.79)	<0.001
Age at the index date	1.10 (1.09, 1.12)	<0.001	1.12 (1.11, 1.13)	<0.001
Sex (female = 1)	0.93 (0.66, 1.29)	0.646	0.59 (0.47, 0.73)	<0.001
Socioeconomic status				
Low	1 (ref)		1 (ref)	
Medium	0.94 (0.68, 1.28)	0.679	0.76 (0.60, 0.96)	0.021
High	0.56 (0.37, 0.87)	0.009	0.65 (0.48, 0.87)	0.004
BMI > 30^a^	1.05 (0.77, 1.42)	0.764	1.12 (0.90, 1.39)	0.328
Diabetes mellitus^a^	1.64 (1.20, 2.24)	0.002	1.43 (1.15, 1.78)	0.001
Hypertension^a^	1.49 (1.06, 2.07)	0.020	1.41 (1.10, 1.81)	0.007
Ever smoking	2.46 (1.76, 3.44)	<0.001	2.0 (1.41, 2.83)	<0.001
Systolic BP^a^	1.0 (0.99, 1.01)	0.868	1.0 (0.99, 1.01)	0.844
LDL-C^a^	0.99 (0.98, 1.01)	0.324	0.99 (0.98, 1.0)	0.144
Total cholesterol	1.0 (0.99, 1.01)	0.951	1.0 (0.99, 1.01)	0.304
Aspirin	0.84 (0.63, 1.12)	0.225	0.98 (0.79, 1.20)	0.819
Beta blockers^a^	1.47 (1.11, 1.96)	0.008	1.38 (1.11, 1.71)	0.003
P2Y12 inhibitors^a^	1.09 (0.82, 1.43)	0.567	1.80 (1.28, 2.54)	<0.001
OSA	0.95 (0.59, 1.54)	0.838	1.06 (0.71, 1.59)	0.773
MDD^a^	1.34 (0.55, 3.31)	0.520	1.96 (1.09, 3.51)	0.024
NAFL^a^	1.09 (0.51, 2.34)	0.825	1.38 (0.75, 2.53)	0.303
LVH^a^	2.37 (1.03, 5.42)	0.042	0.70 (0.17, 2.82)	0.615

Only a few events of death were recorded in the FH group; therefore, this group was not included in the analysis.

Data were analyzed using a multivariable Cox regression model with the low SES set as a reference and female = 1.

ASCVD, patients with atherosclerotic cardiovascular disease; ASCVD-RE, ASCVD-risk equivalent patients; BMI, body mass index; BP, blood pressure; FH, familial hypercholesterolemia; LDL-C, low-density lipoprotein cholesterol; LVH, left ventricular hypertrophy; MDD, major depressive disorder; NAFL, non-alcoholic fatty liver; OR, odds ratio; OSA, obstructive sleep apnea; P2Y12 inhibitors, purinergic receptor P2Y12 protein (G-protein-coupled) inhibitors; PDC, proportion of days covered; SES, socioeconomic status.

a Patient characteristic before the index date.

### 3.5 Healthcare resource utilization

Utilization of healthcare resources during the study period was estimated for patients with PDC ≥ 80% and PDC < 80% across the three patient groups.

#### 3.5.1 Hospitalizations

##### 3.5.1.1 Patients with PDC ≥ 80%

In the FH group, 45 (23.9%) patients experienced at least one hospitalization during the study, with a mean (SD) of 0.27 (1.3) hospitalizations per PY and 1.15 (2.4) hospitalizations per hospitalized patient. The mean (SD) number of hospitalization days per PY was 3.0 (20.9), and the mean (SD) duration of hospitalization per patient was 12.5 (41.7) days.

In the ASCVD group, 1,492 (54.3%) patients experienced at least one hospitalization during the study, with a mean (SD) of 0.48 (1.9) hospitalizations per PY and 0.89 (2.5) hospitalizations per patient. The mean (SD) number of hospitalization days per PY was 2.1 (9.8), and the mean (SD) duration of hospitalization per patient was 3.9 (13.0) days.

In the ASCVD-RE group, 1,360 (42.2%) patients were hospitalized during the study period, with a mean (SD) of 0.32 (1.7) hospitalizations per PY and 0.75 (2.6) hospitalizations per patient. The mean (SD) number of hospitalization days per PY was 1.5 (8.2), and the mean (SD) duration of hospitalization per patient was 3.7 (12.2) days.

##### 3.5.1.2 Patients with PDC < 80%

In the FH group, all hospitalization-related parameters were lower in patients with PDC < 80% than in those with PDC ≥ 80%. Overall, the number of patients with any hospitalization, the mean number of hospitalizations per PY and per patient, and the duration of hospitalizations per PY and per patient were similar between patients with PDC ≥ 80% and PDC < 80% in the ASCVD and ASCVD-RE groups.

Within-group differences for all hospitalization-related parameters were not statistically significant, except for the total number of hospitalizations in the ASCVD-RE group (*p* = 0.015).

#### 3.5.2 Emergency room visits

In the FH group, patients with PDC ≥ 80% had somewhat higher mean (SD) numbers of ER visits than those with PDC < 80% [0.43 (1.4) vs. 0.28 (0.6)]. Similarly, the mean (SD) number of ER visits per patient was 1.05 (2.0) among patients with PDC ≥ 80% and 0.59 (0.8) among those with PDC < 80%.

The number of ER visits during the study period was similar between patients with PDC ≥ 80% and those with PDC < 80% in both the ASCVD group [0.77 (7.2) vs. 0.70 (1.3)] and the ASCVD-RE group [0.43 (1.8) vs. 0.40 (0.9)]. However, in both groups, the mean (SD) number of ER visits per patient was notably higher for patients with PDC ≥ 80% [1.21 (9.0) and 0.84 (2.5) in the ASCVD and ASCVD-RE groups, respectively] than for those with PDC < 80% [1.02 (1.4) and 0.76 (1.1) in the ASCVD and ASCVD-RE groups, respectively].

The within-group differences for all ER assessments were not statistically significant, except for ER visits per patient in the FH group (*p* = 0.046).

#### 3.5.3 Primary care physician and cardiologist visits

Overall, across all three study groups, the number of PCP and cardiologist visits per PY was higher among patients with PDC ≥ 80% than among those with PDC < 80%.

Among patients with PDC ≥ 80%, the mean (SD) number of PCP visits per PY was highest in the ASCVD group [14.6 (10.9)], followed by the ASCVD-RE group [12.7 (7.6)] and the FH group [11.4 (7.3)]. The number of patients who visited a cardiologist was highest in the ASCVD group [1,948 (70.9%)], followed by 1,132 (35.1%) patients in the ASCVD-RE group and 34 (18.1%) patients in the FH group.

Similarly, among patients with PDC < 80%, the highest mean (SD) number of PCP visits per PY was recorded in the ASCVD group [12.1 (8.7)], followed by the ASCVD-RE group [10.2 (7.7)] and the FH group [8.6 (8.1)]. Consistent with these observations, cardiologist visits were recorded for 1,339 (57.4%) patients in the ASCVD group, 1,575 (26.7%) patients in the ASCVD-RE group, and 121 (13.8%) patients in the FH group.

All within-group differences for PCP visits across the three study groups were statistically significant (*p* < 0.001). Differences in cardiologist visits, were also statistically significant (*p* < 0.001) for both the ASCVD and ASCVD-RE groups but not for the FH group.

## 4 Discussion and overall conclusions

In this large retrospective real-world database study, several important findings emerged regarding treatment patterns, adherence to lipid-lowering therapy, LDL-C control, clinical outcomes, and healthcare resource utilization among treated high-risk patients with hypercholesterolemia in Israel.

The baseline characteristics of the study population were broadly consistent with the expected clinical profiles of patients with ASCVD, ASCVD risk-equivalent conditions, and FH. As expected, baseline LDL-C, total cholesterol, and triglyceride levels were highest in the FH group. Initial treatment was predominately statin monotherapy, whereas the use of combination lipid-lowering regimens was limited.

Among patients with high adherence (PDC ≥ 80%), combination therapy with HIS + ezetimibe was associated with the most favorable LDL-C patterns, particularly in the ASCVD and ASCVD-RE groups. In contrast, the small number of patients receiving PCSK9 inhibitors may reflect limited accessibility of these medications during the study period, which should be considered when interpreting the treatment pattern findings.

Adherence to the five main LLT categories was highest in the ASCVD group, intermediate in the ASCVD-RE group, and lowest in the FH group. Higher adherence was associated with a greater likelihood of achieving controlled LDL-C levels within the first months after treatment initiation. In particular, HIS + ezetimibe was associated with the highest proportion of patients achieving LDL-C <55 mg/dL in the ASCVD and ASCVD-RE groups, whereas non-HIS-based regimens were generally associated with less pronounced LDL-C lowering.

Across the five main LLT categories, LDL-C patterns remained broadly stable over time among patients with PDC ≥ 80%. Mean LDL-C levels among adherent patients were <70 mg/dL in the ASCVD group and <100 mg/dL in the ASCVD-RE group but remained >100 mg/dL in the FH group. These findings are consistent with the LDL-C targets recommended during the study period for ASCVD and ASCVD-RE but not for FH (19). This distinction is important because LDL-C targets have become more stringent in recent years (11), and the present findings should therefore be interpreted in the context of the guideline recommendations that applied during 2013–2019. Overall, patients with FH were less likely to achieve LDL-C targets, even with high adherence, highlighting the greater therapeutic challenge posed by this population during the study period.

PDC ≥ 80% was significantly associated with controlled LDL-C levels in both the ASCVD and ASCVD-RE groups, consistent with previous retrospective studies linking statin persistence with improved lipid control (10). In multivariable analyses, several demographic and clinical characteristics were also associated with LDL-C control, although the specific patterns differed between study groups. In the FH group, few variables were significantly associated with LDL-C control, likely reflecting the smaller number of patients who achieved target LDL-C levels in this population.

These findings are consistent with previous real-world studies demonstrating that adherence to lipid-lowering therapy is an important determinant of lipid control in routine clinical practice (8, 10).

PDC ≥ 80% was not significantly associated with time to first MI, CVA, or TIA in the ASCVD-RE group. However, higher adherence was associated with a longer time to coronary revascularization (PCI or CABG) in both the ASCVD and ASCVD-RE groups.

The number and duration of hospitalizations, as well as emergency room visits, were generally similar between patients with PDC ≥ 80% and those with PDC < 80% in the ASCVD and ASCVD-RE groups. In the FH group, hospitalization-related measures were somewhat higher among patients with PDC ≥ 80%. Across all study groups, patients with higher adherence had more visits to primary care physicians and cardiologists. These findings may reflect greater engagement with healthcare services and closer clinical follow-up among adherent patients, rather than a direct causal effect of adherence on healthcare utilization.

Another methodological consideration relates to the estimation of LDL-C levels. In this study, LDL-C values were calculated using the Friedewald equation as part of routine laboratory reporting within Maccabi Healthcare Services. Although this approach has been widely used in both clinical practice and epidemiological studies, previous studies have shown that calculated LDL-C values may differ from directly measured LDL-C, particularly at low LDL-C levels or in patients with elevated triglycerides (23–25). These discrepancies may affect classification around clinically relevant treatment thresholds, such as 100, 70, and 55 mg/dL (24–26). Although triglyceride levels in the cohort were generally within ranges in which the Friedewald equation is commonly applied, some degree of misclassification around clinically relevant LDL-C thresholds cannot be excluded and should be considered when interpreting the LDL-C goal attainment analyses.

This study has several limitations. First, the cohort included only treated patients and may therefore underestimate the broader burden of uncontrolled hypercholesterolemia in the underlying source population. Second, the FH group included both confirmed and probable FH cases, which may have introduced clinical heterogeneity (13, 18). Third, cardiovascular risk classification in the ASCVD-RE group was based on the SCORE system rather than SCORE2, which became available after the study period (19, 27, 28).

Because adherence was assessed at the treatment-course level, some patients could contribute more than one treatment episode during the study period, which may have introduced within-patient correlation.

As in other observational studies, several additional sources of bias should be considered. Patients with higher adherence may differ systematically from less adherent patients in ways not fully captured in the available data (healthy adherer bias). In addition, treatment intensification may reflect underlying disease severity or physician decision-making, introducing potential confounding by indication. Because adherence was assessed over time, time-related biases such as immortal time bias cannot be completely excluded. Finally, differences in the intensity of clinical follow-up and the frequency of laboratory testing may have influenced LDL-C monitoring and outcome detection.

In this real-world cohort, substantial gaps in LDL-C control were observed across high-risk populations, particularly among patients with FH. Higher adherence to lipid-lowering therapy was consistently associated with improved LDL-C control and more favorable cardiovascular outcome patterns in selected analyses.

In conclusion, this real-world study identified substantial gaps in LDL-C control and treatment adherence among treated high-risk patients with hypercholesterolemia in Israel, with the greatest unmet need observed among patients with FH. Higher adherence to lipid-lowering therapy was consistently associated with improved LDL-C control and with more favorable cardiovascular outcome patterns in selected analyses; however, these findings should be interpreted as associations rather than evidence of causality due to the observational study design.

## Data Availability

The original contributions presented in the study are included in the article/Supplementary Material, further inquiries can be directed to the corresponding author.
