# Supplementary material for: Treatment patterns and adherence to lipid-lowering therapy, LDL-C control, clinical outcomes, and healthcare resource utilization in treated patients with hypercholesterolemia at high cardiovascular risk in Israel: a retrospective database study
Source: Front Cardiovasc Med. 2026 Apr 30;13:1672698. doi: 10.3389/fcvm.2026.1672698 (PMC13171751; doi:10.3389/fcvm.2026.1672698)
Supplement: Supplementary file 1 [file Datasheet1.pdf]

11. Supplementary Data

Table 1a. Selection of Study Population

Attrition criterion	Patients excluded	Number of patients
Number of patients with first LLT purchase between 2013-2019		118,449
Number of patients whose first LLT purchase was statin	10,400	108,049
Number of patients >18 years of age	388	107,661
Number of patients in cancer registry	12,354	95,307
Number of patients with continuous enrolment in MHS one year prior to index date	20,320	**74,987**

Table 1b. First LLT Medications Used in the Study Period

LLT Medication	N (%)
**ONLY statins**	**108,049 (91.2%)**
Fibrates	5521 (4.7%)
Bile acid sequestrants	619 (0.5%)
Ezetimibe	846 (0.7%)
PCSK9 inhibitors	21 (0.0%)
LLT combinations	3393 (2.9%)
**Total**	**118,449 (100%)**

LLT = lipid=lowering therapy; PCSK9 INHIBITORS = proprotein convertase subtilisin kexin type 9 inhibitor

Table 2. Study Groups

Eligible population (N=74,987)		Without LDL in the year before index (treatment initiation)	Final study population with LDL in the year before index*
Number of **FH** patients	1344		
Number of FH patients after exclusion, if notes (1) and (2) are accepted	1116	54	**1062**
Number of **ASCVD** patients	8022	2939	**5083**
Number of **ASCVD-RE** patients	9583	470	**9113**
**Number of patients in the cohort**	18,721		**15,258**

ASCVD = patients with atherosclerotic cardiovascular disease; ASCVD-RE = ASCVD-risk equivalent patients; FH = familial hypercholesterolemia; LDL = low-density lipoprotein

Note: (1) FH patients remaining after exclusion according to diagnoses = 1148 individuals; (2) Excluded patients diagnosed >65 years by MED_PED age≥40 = 32 individuals.

Note: The excluded 32 individuals were added to the eligible populations in the ASCVD and ASCVD-RE groups.

Table 3. Adherence to LLT Medications by Study Group, LLT Type, and Statin Intensity

LLT	PDC	FH n=1062	ASCVD n=5083	ASCVD-RE n=9113	**P value** between main 3 groups, PDC≥80 and PDC<80
HIS monotherapy	PDC≥80	89 (22.3%)	2082 (65.1%)	958 (46.8%)	
	60≤PDC<80	48 (12.0%)	326 (10.2%)	213 (10.4%)	
	40≤PDC<60	41 (10.3%)	243 (7.6%)	174 (8.5%)	
	20≤PDC<40	70 (17.5%)	259 (8.1%)	235 (11.5%)	
	PDC<20	151 (37.8%)	287 (9.0%)	467 (22.8%)	
	All	399	3197	2047	<0.001
Non-HIS monotherapy	PDC≥80	142 (17.4%)	1261 (43.7%)	2735 (33.9%)	
	60≤PDC<80	68 (8.3%)	346 (12.0%)	862 (10.7%)	
	40≤PDC<60	100 (12.3%)	279 (9.7%)	881 (10.9%)	
	20≤PDC<40	125 (15.3%)	355 (12.3%)	1032 (12.8%)	
	PDC<20	380 (46.6%)	645 (22.3%)	2554 (31.7%)	
	All	815	2886	8064	<0.001
Ezetimibe monotherapy	PDC≥80	9 (52.9%)	62 (50.8%)	94 (45.9%)	
	60≤PDC<80	3 (17.6%)	18 (14.8%)	24 (11.7%)	
	40≤PDC<60	0	6 (4.9%)	15 (7.3%)	
	20≤PDC<40	3 (17.6%)	17 (13.9%)	24 (11.7%)	
	PDC<20	2 (11.8%)	19 (15.6%)	48 (23.4%)	
	All	17	122	205	0.627
HIS+ezetimibe	PDC≥80	43 (53.8%)	387 (80.6%)	176 (73.9%)	
	60≤PDC<80	10 (12.5%)	36 (7.5%)	14 (5.9%)	
	40≤PDC <60	13 (16.3%)	25 (5.2%)	17 (7.1%)	
	20≤PDC <40	9 (11.3%)	14 (2.9%)	16 (6.7%)	
	PDC <20	5 (6.3%)	18 (3.8%)	15 (6.3%)	
	All	80	480	238	<0.001
Non-HIS+ezetimibe	PDC≥80	35 (52.2%)	257 (66.1%)	255 (60.6%)	
	60≤PDC<80	8 (11.9%)	39 (10.0%)	37 (8.8%)	
	40≤PDC<60	7 (10.4%)	37 (9.5%)	38 (9.0%)	
	20≤PDC<40	9 (13.4%)	31 (8.0%)	43 (10.2%)	
	PDC<20	8 (11.9%)	25 (6.4%)	48 (11.4%)	
	All	67	389	421	0.056 for ASCVD

**LLT**	**PDC**	**FH n=1062**	**ASCVD n=5083**	**ASCVD-RE n=9113**	**P value** between main 3 groups, PDC≥80 and PDC<80
					vs. ASCVD-RE: 0.105
PCSK9 inhibitors monotherapy	PDC≥80	6 (75.0%)	38 (67.9%)	13 (59.1%)	
	60≤PDC<80	1 (12.5%)	7 (12.5%)	0	
	40≤PDC<60	0	4 (7.1%)	2 (9.1%)	
	20≤PDC 40	1 (12.5%)	1 (1.8%)	1 (4.5%)	
	PDC <20	0	6 (10.7%)	6 (27.3%)	
	All	8	56	22	
PCSK9 inhibitors+statin	PDC≥80	3 (100%)	13 (100%)		
	60≤PDC<80				
	40≤PDC<60				
	20≤PDC<40				
	PDC<20				
	All	3	13	0	
PCSK9 inhibitors+ezetimibe	PDC≥80	2 (100%)	3 (100%)	2 (100%)	
Fibrates	PDC≥80	10 (40.0%)	11 (68.8%)	18 (37.5%)	
	60≤PDC<80	3 (12.0%)	1 (6.3%)	4 (8.3%)	
	40≤PDC<60	0	0	7 (14.6%)	
	20≤PDC<40	4 (16.0%)	3 (18.8%)	7 (14.6%)	
	PDC<20	8 (32.0%)	1 (6.3%)	12 (25.0%)	
	All	25	16	48	
Fibrates+statin	PDC≥80	23 (50.0%)	23 (69.7%)	27 (60.0%)	
	60≤PDC<80	7 (15.2%)	2 (6.1%)	5 (11.1%)	
	40≤PDC<60	5 (10.9%)	3 (9.1%)	7 (15.6%)	
	20≤PDC<40	6 (13.0%)	1 (3.0%)	4 (8.9%)	
	PDC<20	5 (10.9%)	4 (12.1%)	2 (4.4%)	
	All	46	33	45	

ASCVD = patients with atherosclerotic cardiovascular disease; ASCVD-RE = ASCVD-risk equivalent patients; FH = familial hypercholesterolemia; HIS = high intensity statin; LLT = lipid lowering therapy; NHIS = non high intensity statin; PDC = proportion of days covered; PSCK9i = proprotein convertase subtilisin kexin type 9 inhibitor

Table 4. First LDL after Treatment Initiation* (PDC≥80%)

First LDL after treatment initiation	Patients with LDL-C measurement	FH n=1062	ASCVD n=5083	ASCVD-RE n=9113
HIS monotherapy	**PDC≥80**	**89 (8.4%)**	**2082 (40.9%)**	**958 (10.5%)**
	LDL, mean (SD)	n=52<br>117.0 (47.2)	n=1679<br>62.0 (22.8)	n=644<br>71.7 (27.9)
	LDL<55, n (%)	5 (9.6%)	700 (41.7%)	184 (28.6%)
	LDL 55-70	1 (1.9%)	486 (28.9%)	169 (26.2%)
	LDL 70-100	16 (30.8%)	404 (24.1%)	204 (31.7%)
	LDL≥100	30 (57.7%)	89 (5.3%)	87 (13.5%)
Non-HIS monotherapy	**PDC≥80**	**142 (17.4%)**	**1261 (43.7%)**	**2735 (33.9%)**
	LDL, mean (SD)	n=96<br>121.1 (45.3)	n=872<br>72.3 (23.3)	n=1792<br>86.5 (29.0)
	LDL<55, n (%)	2 (2.1%)	191 (21.9%)	190 (10.6%)
	LDL 55-70	5 (5.2%)	261 (29.9%)	358 (20.0%)
	LDL 70-100	28 (29.2%)	314 (36.0%)	769 (42.9%)
	LDL≥100	61 (63.5%)	106 (12.2%)	475 (26.5%)
Ezetimibe monotherapy	**PDC≥80**	**9 (52.9%)**	**62 (50.8%)**	**94 (45.9%)**
	LDL, mean (SD)	n=2<br>146.9 (35.2)	n=36<br>91.8 (32.8)	n=52<br>103.6 (33.1)
	LDL<55, n (%)	-	3 (8.3%)	3 (5.8%)
	LDL 55-70	-	9 (25.0%)	4 (7.7%)
	LDL 70-100	-	10 (27.8%)	19 (36.5%)
	LDL≥100	2	14 (38.9%)	26 (50.0%)
HIS+ezetimibe	**PDC≥80**	**43 (53.8%)**	**387 (80.6%)**	**176 (73.9%)**
	LDL, mean (SD)	n=17<br>96.5 (40.3)	n=227<br>59.6 (23.7)	n=98<br>61.6 (31.2)
	LDL<55, n (%)	2 (11.8%)	108 (47.6%)	51 (52.0%)
	LDL 55-70	3 (17.6%)	69 (30.4%)	28 (28.6%)
	LDL 70-100	5 (29.4%)	35 (15.4%)	7 (7.1%)
	LDL≥100	7 (41.2%)	15 (6.6%)	12 (12.2%)
Non-HIS+ezetimibe	**PDC≥80**	**35 (52.2%)**	**257 (66.1%)**	**255 (60.6%)**
	LDL, mean (SD)	n=19<br>133.0 (64.5)	n=158<br>64.5 (26.4)	n=143<br>75.0 (35.2)
	LDL<55, n (%)	0	68 (43.0%)	41 (28.7%)
	LDL 55-70	3 (15.8%)	44 (27.8%)	36 (25.2%)
	LDL 70-100	6 (31.6%)	33 (20.9%)	42 (29.4%)
	LDL≥100	10 (52.6%)	13 (8.2%)	24 (16.8%)

ASCVD = patients with atherosclerotic cardiovascular disease; ASCVD-RE = ASCVD-risk equivalent patients; FH = familial hypercholesterolemia; HIS = high intensity statin; LDL = low-density lipoprotein; LLT = lipid lowering

therapy; NHIS = non high intensity statin; PDC = proportion of days covered; PSCK9i = proprotein convertase subtilisin kexin type 9 inhibitor
*Within 3 to 6 months from treatment initiation, when LDL-C could be measured.

Table 5. LDL-C Levels During Each LLT Therapy (PDC≥80%)

Mean LDL-C after treatment initiation	Patients with LDL-C measurement	FH n=1062	ASCVD n=5083	ASCVD-RE n=9113
HIS monotherapy	**PDC≥80**	**89**	**2082**	**958**
	LDL, mean (SD)	n=69<br>111.7 (46.8)	n=2006<br>63.9 (20.4)	n=875<br>70.9 (24.5)
	LDL<55, n (%)	8 (11.6%)	699 (34.8%)	230 (26.3%)
	LDL 55-70	3 (4.3%)	659 (32.9%)	239 (27.3%)
	LDL 70-100	19 (27.5%)	555 (27.7%)	313 (35.8%)
	LDL≥100	39 (56.5%)	93 (4.6%)	93 (10.6%)
Non-HIS monotherapy	**PDC≥80**	**142**	**1261**	**2735**
	LDL, mean (SD)	n=121<br>119.8 (35.7)	n=1191<br>73.3 (20.8)	n=2607<br>84.1 (23.7)
	LDL<55, n (%)	1 (0.8%)	218 (18.3%)	222 (8.5%)
	LDL 55-70	6 (5.0%)	363 (30.5%)	520 (19.9%)
	LDL 70-100	30 (24.8%)	485 (40.7%)	1281 (49.1%)
	LDL≥100	84 (69.4%)	125 (10.5%)	584 (22.4%)
Ezetimibe monotherapy	**PDC≥80**	**9**	**62**	**94**
	LDL, mean (SD)	n=4<br>152.1 (8.1)	n=41<br>94.5 (25.8)	n=65<br>109.3 (30.1)
	LDL<55, n (%)		1 (2.4%)	1 (1.5%)
	LDL 55-70		9 (22.0%)	3 (4.6%)
	LDL 70-100		14 (34.1%)	22 (33.8%)
	LDL≥100	4	17 (41.5%)	39 (60.0%)
HIS+ezetimibe	**PDC≥80**	**43**	**387**	**176**
	LDL, mean (SD)	n=25<br>101.7 (38.8)	n=297<br>59.7 (19.9)	n=135<br>64.5 (28.9)
	LDL<55, n (%)	2 (8.0%)	135 (45.5%)	57 (42.2%)
	LDL 55-70	3 (12.0%)	91 (30.6%)	42 (31.1%)
	LDL 70-100	11 (44.0%)	58 (19.5%)	23 (17.0%)
	LDL≥100	9 (36.0%)	13 (4.4%)	13 (9.6%)
Non-HIS+ezetimibe	**PDC≥80**	**35**	**257**	**255**
	LDL, mean (SD)	n=26<br>119.5 (49.4)	n=193<br>65.5 (22.4)	n=189<br>73.7 (29.9)
	LDL<55, n (%)	0	68 (35.2%)	47 (24.9%)
	LDL 55-70	5 (19.2%)	58 (30.1%)	59 (31.2%)

Mean LDL-C after treatment initiation	Patients with LDL-C measurement	FH n=1062	ASCVD n=5083	ASCVD-RE n=9113
	LDL 70-100	6 (23.1%)	56 (29.0%)	48 (25.4%)
	LDL≥100	15 (57.7%)	11 (5.7%)	35 (18.5%)

ASCVD = patients with atherosclerotic cardiovascular disease; ASCVD-RE = ASCVD-risk equivalent patients; FH = familial hypercholesterolemia; HIS = high intensity statin; LDL = low-density lipoprotein; LLT = lipid lowering therapy; NHIS = non high intensity statin; PDC = proportion of days covered; PSCK9i = proprotein convertase subtilisin kexin type 9 inhibitor

Table 6. LDL-C Levels During Each LLT Therapy (PDC<80%)

Mean LDL-C after treatment initiation	Patients with LDL-C measurement	FH n=1062	ASCVD n=5083	ASCVD-RE n=9113
HIS monotherapy	**PDC<80**	**310**	**1115**	**1089**
	LDL, mean (SD)	n=254<br>159.0 (58.8)	n=1060<br>93.6 (31.8)	n=989<br>113.0 (37.4)
	LDL<55, n (%)	10 (3.9%)	93 (8.8%)	48 (4.9%)
	LDL 55-70	9 (3.5%)	177 (16.7%)	72 (7.3%)
	LDL 70-100	17 (6.7%)	383 (36.1%)	263 (26.6%)
	LDL≥100	218 (85.8%)	407 (38.4%)	606 (61.3%)
Non-HIS monotherapy	**PDC<80**	**673**	**1625**	**5329**
	LDL, mean (SD)	n=592<br>160.5 (45.7)	n=1555<br>105.8 (30.5)	n=5028<br>119.4 (31.4)
	LDL<55, n (%)	6 (1.0%)	50 (3.2%)	75 (1.5%)
	LDL 55-70	3 (0.5%)	124 (8.0%)	181 (3.6%)
	LDL 70-100	32 (5.4%)	510 (32.8%)	1166 (23.2%)
	LDL≥100	551 (93.1%)	871 (56.0%)	3606 (71.7%)
Ezetimibe monotherapy	**PDC<80**	**8**	**60**	**111**
	LDL, mean (SD)	n=8<br>144.2 (24.3)	n=52<br>123.2 (29.2)	n=90<br>132.3 (32.6)
	LDL<55, n (%)		1 (1.9%)	0
	LDL 55-70		1 (1.9%)	1 (1.1%)
	LDL 70-100		9 (17.3%)	15 (16.7%)
	LDL≥100	8	41 (78.8%)	74 (82.2%)
HIS+ezetimibe	**PDC<80**	**37**	**93**	**62**
	LDL, mean (SD)	n=25<br>150.1 (59.7)	n=70<br>98.9 (37.3)	n=53<br>105.5 (40.7)
	LDL<55, n (%)	0	11 (15.7%)	6 (11.3%)
	LDL 55-70	3 (12.0%)	6 (8.6%)	3 (5.7%)
	LDL 70-100	3 (12.0%)	17 (24.3%)	18 (34.0%)
	LDL≥100	19 (76.0%)	36 (51.4%)	26 (49.1%)
Non-HIS+ezetimibe	**PDC<80**	**32**	**132**	**166**
	LDL, mean (SD)	n=25<br>145.9 (54.6)	n=108<br>102.4 (34.0)	n=131<br>116.4 (38.5)
	LDL<55, n (%)	1 (4.0%)	8 (7.4%)	7 (5.3%)
	LDL 55-70	1 (4.0%)	16 (14.8%)	10 (7.6%)
	LDL 70-100	4 (16.0%)	28 (25.9%)	28 (21.4%)
	LDL≥100	19 (76.0%)	56 (51.9%)	86 (65.6%)

ASCVD = patients with atherosclerotic cardiovascular disease; ASCVD-RE = ASCVD-risk equivalent patients; FH = familial hypercholesterolemia; HIS = high intensity statin; LDL = low-density lipoprotein; LLT = lipid lowering

therapy; NHIS = non high intensity statin; PDC = proportion of days covered; PSCK9i = proprotein convertase subtilisin kexin type 9 inhibitor

Table 7. Factors Associated with the Time to PCI and CABG Procedures After LLT Initiation for ASCVD and ASCVD-RE Groups

Patient characteristics	ASCVD n=5083		ASCVD-RE n=9113	
	HR (95% CI)	p-value	HR (95% CI)	p-value
PDC≥80%	0.79 (0.63, 0.99)	0.045	0.75 (0.57, 0.99)	0.044
Age at index date	0.99 (0.98, 1.0)	0.195	1.02 (1.01, 1.03)	0.004
Sex (female=1)	0.42 (0.31, 0.59)	<0.001	0.24 (0.17, 0.33)	<0.001
Socioeconomic status				
Low	1 (ref)		1 (ref)	
Medium	0.99 (0.75, 1.31)	0.968	0.87 (0.63, 1.19)	0.374
High	1.0 (0.72, 1.39)	1.00	1.01 (0.70, 1.46)	0.947
BMI* >30	1.25 (0.98, 1.58)	0.067	0.96 (0.73, 1.27)	0.764
Diabetes mellitus*	1.39 (1.04, 1.86)	0.028	1.68 (1.27, 2.22)	<0.001
Hypertension*	1.07 (0.84, 1.36)	0.580	0.98 (0.74, 1.31)	0.907
Ever smoking	1.25 (0.96, 1.61)	0.092	0.85 (0.52, 1.40)	0.520
Systolic BP*	1.0 (0.99, 1.01)	0.621	1.01 (1.01, 1.02)	<0.001
LDL-C*	0.99 (0.99, 1.01)	0.376	1.0 (0.99, 1.01)	0.128
Total cholesterol	1.01 (0.99, 1.01)	0.240	1.0 (0.99, 1.01)	0.211
Aspirin	0.88 (0.70, 1.11)	0.287	0.96 (0.72, 1.27)	0.767
Beta blockers*	1.50 (1.19, 1.89)	<0.001	1.36 (1.0, 1.84)	0.047
P2Y12 inhibitors*	1.56 (1.23, 1.97)	<0.001	2.46 (1.53, 3.95)	<0.001
OSA	0.92 (0.64, 1.32)	0.630	0.95 (0.58, 1.53)	0.819
MDD*	0.61 (0.23, 1.63)	0.323	1.15 (0.47, 2.81)	0.754
NAFL*	0.73 (0.34, 1.56)	0.419	0.86 (0.35, 2.10)	0.743

ASCVD = patients with atherosclerotic cardiovascular disease; ASCVD-RE = ASCVD-risk equivalent patients; BMI = body mass index; BP = blood pressure; FH = familial hypercholesterolemia; LDL-C = low-density lipoprotein cholesterol; MDD = major depressive disorder; NAFL = nonalcoholic fatty liver; HR = hazard ratio; OSA = obstructive sleep apnea; P2Y12 inhibitors = purinergic receptor P2Y, G-protein coupled, 12 protein inhibitors; PDC = proportion of days covered; SES = socioeconomic status.
*Patient characteristic before index date.
Note: Only a few events were recorded in the FH group; therefore, this group was not included in the analysis.
Data analyzed by multivariable Cox regression model with the low SES set as a reference and female=1.

Appendix A: ATC/IC-9 Codes and Registries

Generic	ATC	
**Statins**	C10AA05	atorvastatin
**(by statin intensity)**	C10BX08	atorvastatin and acetylsalicylic acid
	C10BX03	atorvastatin and amlodipine
	C10BA05	atorvastatin and ezetimibe
	C10BA08	atorvastatin and omega-3 fatty acids
	C10BX15	atorvastatin and perindopril
	C10BX12	atorvastatin, acetylsalicylic acid and perindopril
	C10BX06	atorvastatin, acetylsalicylic acid and ramipril
	C10BX11	atorvastatin, amlodipine and perindopril
	C10BX18	atorvastatin, amlodipine and ramipril
	C10AA06	cerivastatin
	C10AA04	fluvastatin
	A10BH52	gemigliptin and rosuvastatin
	C10AA02	lovastatin
	C10BA01	lovastatin and nicotinic acid
	L01XX08	pentostatin
	C10AA08	pitavastatin
	C10AA03	pravastatin
	C10BX02	pravastatin and acetylsalicylic acid
	C10BA03	pravastatin and fenofibrate
	C10AA07	rosuvastatin
	C10BX05	rosuvastatin and acetylsalicylic acid
	C10BX09	rosuvastatin and amlodipine
	C10BA06	rosuvastatin and ezetimibe
	C10BA09	rosuvastatin and fenofibrate
	C10BX16	rosuvastatin and fimasartan
	C10BA07	rosuvastatin and omega-3 fatty acids
	C10BX17	rosuvastatin and ramipril
	C10BX10	rosuvastatin and valsartan
	C10BX07	rosuvastatin, amlodipine and lisinopril
	C10BX14	rosuvastatin, amlodipine and perindopril
	C10BX13	rosuvastatin, perindopril and indapamide
	C10AA01	simvastatin
	C10BX01	simvastatin and acetylsalicylic acid

Generic	ATC	
	C10BA02	simvastatin and ezetimibe
	C10BA04	simvastatin and fenofibrate
	C10BX04	simvastatin, acetylsalicylic acid and ramipril
	A10BH51	sitagliptin and simvastatin
	H01CB01	somatostatin
	B02AB05	ulinastatin
**PCSK9 inhibitors**	C10AX14, C10AX13	
**Ezetimibe**	C10BA05	atorvastatin and ezetimibe
	C10BA10	bempedoic acid and ezetimibe
	C10AX09	ezetimibe
	C10BA06	rosuvastatin and ezetimibe
	C10BA02	simvastatin and ezetimibe
**Fibrates (C10AB)**	C10AB01	clofibrate
	C10AB02	bezafibrate
	C10AB03	aluminium clofibrate
	C10AB04	gemfibrozil
	C10AB05	fenofibrate micronised
	C10AB06	simfibrate
	C10AB07	ronifibrate
	C10AB08	ciprofibrate
	C10AB09	etofibrate
	C10AB10	clofibride
	C10AB11	choline fenofibrate
**Bile acid sequestrants (C10AC)**	C10AC01	colestyramine
	C10AC02	colestipol
	C10AC03	colextran
	C10AC04	colesevelam
**Mipomersen**	C10AX11	
**Lomitapide**	C10AX12	
**Beta blockers**	C07BB04	acebutolol (Sectral)
	C07AB03	atenolol (Tenormin)
	C07AB01	practolol
	C07AB02	metoprolol
	C07AB03	atenolol
	C07AB04	acebutolol

Generic	ATC	
	C07AB05	betaxolol
	C07AB06	bevantolol
	C07AB07	bisoprolol
	C07AB08	celiprolol
	C07AB09	esmolol
	C07AB10	epanolol
	C07AB11	s-atenolol
	C07AB12	nebivolol
	C07AB13	talinolol
	C07AB14	landiolol
**Angiotensin converter enzyme (ACE) inhibitors**	C09AA01	captopril
	C09AA02	enalapril
	C09AA03	lisinopril
	C09AA04	perindopril
	C09AA05	ramipril
	C09AA06	quinapril
	C09AA07	benazepril
	C09AA08	cilazapril
	C09AA09	fosinopril
	C09AA10	trandolapril
	C09AA11	spirapril
	C09AA12	delapril
	C09AA13	moexipril
	C09AA14	temocapril
	C09AA15	zofenopril
	C09AA16	imidapril
**P2Y12 inhibitors**	B01AC04	clopidogrel
	B01AC22	prasugrel
	B01AC24	ticagrelor
	B01AC25	cangrelor
**Aldosterone antagonists**	C03DA01	spironolactone
	C03DA02	potassium canrenoate
	C03DA03	canrenone
	C03DA04	eplerenone
**Angiotensin II receptor blockers**	C09CA01	losartan
	C09CA02	eprosartan

Generic	ATC	
	C09CA03	valsartan
	C09CA04	irbesartan
	C09CA05	tasosartan
	C09CA06	candesartan
	C09CA07	telmisartan
	C09CA08	olmesartan medoxomil
	C09CA09	azilsartan medoxomil
	C09CA10	fimasartan
**Acetylsalicylic acid (ASA)**	A01AD05	aspirin
	B01AC06	aspirin
**SGLT-2 inhibitors**	A10BK01	dapagliflozin
	A10BK02	canagliflozin
	A10BK03	empagliflozin
	A10BK04	ertugliflozin
	A10BK05	ipragliflozin
	A10BK06	sotagliflozin
